# 

**Published:** 1883-08

**Authors:** 


### INDEX.

### A.

PAGE.
Albumen, Picric Acid as a Test for, 510
American Medical Association.... 565
Analysis of Beef Peptonoids. John Attfield ........................ 512
Amputation of Leg. U. C. Lynde, M. D.......................... 400
Aneurism of Thoracic Aorta. A. M. Barker, M. D............... 451
Action of Quinine upon the Ear... 30
Albuminuria .................... 31
Autopsies, Interesting. Frederick Peterson, M. D.....55, 105, 154, 216
Aneurism of Arch of the Aorta—Autopsy. By Frank Dudley Beane, M. D.................. 161
Apoplexy, Complicating Cirrhosis of the Kidneys. James H. Hutchinson, M. D................... 97
Abscess, Cervical. F. R. Campbell, M. D.......................... 221
Abscess of Neck, Treatment of... 313
Anæsthesia in Diseases of the Kidneys—Danger................. 316
Angina Pectoris. a Case of. P. W. Van Peyma, M. D.............. 350
Axis-Traction Forceps............ 253
Autopsy in a Case of Prolonged Vomiting....... ............ 275

### B.

Banta, Dr. R. L. Puerperal Fever, 488
Beane, Frank Dudley, A. M., M. D. The Deafness of Pregnancy and the Post Parturient State... 495
PAGE
Beane, Frank Dudley, A. M., M. D. An Experience with Cannabis Indica......................... 445
Buffalo Medical College.......... 40
Buffalo General Hospital........ 42
Beane, Frank Dudley, A. M., M. D. Aneurism of the Arch of the Aorta—Autopsy ............... 16
Buffalo General Hospital—Surgical Cases Treated. Charles C. F. Gay, M. D.................... 112
Bellevue Hospital Medical College and the Code of the Fathers... 325
Buffalo Obstetrical Society........ 377
Business Change ................ 140

### C.

Causes of Error in Reports Relative to the Physical Evidence of Assaults on Young Girls........ 514
Correction, A. Dr. Storck....... 519
Cholera, the Approach and Prevention of..................... 44
Catarrh of the Naso-Pharynx, or American Catarrh.............. 65
College and the Hospital......... 91
Club Foot. Herman E. Hayd, M. D., M. R. C. S................. 145
Correspondence..........173, 519, 560
Cholera at Allahabad............. 180
Circumcision, Improved Method of, 135
Campbell, F. R., M. D. A Case of Cervical Abscess ........... 221
Case of Extra-Uterine Pregnancy; Death—Autopsy. Edward Clark, M. D......................... 299

PAGE.

Commencement, Thirty-eighth Annual, of the Medical Department of University of Buffalo........ 369
Chloral Poisoning................ 277
Crego, Floyd S., M. D. Epilepsy, 531

### D.

Deafness of Pregnancy and the Post Parturient State. Frank Dudley Beane, A. M., M. D... 495
Diseases, Functional and Structural, 519
Dislocation, Congenital, of Both Hip Joints. Herman Mynter, M. D........................ 406
Diabetes, Puerperal.............. 18
Diarrhœa, Eserine in............. 19
Davidson, A. R., M. D., Pyelo-Nephritis, with Calculi in the Bladder...................... 166
Diphtheria and Scarlatina......... 178
Danger of Glazed Earthenware Vessels...................... 228
Dysentery. E. T. Dorland, M. D............................ 289
Deep Cervical Abscess of the Post Cervical Region, with Sudden Death. B. G. Long, M. D..... 301
Diphtheria...................... 267
Diagnostic Value of Uterine Hemorrhage after the Menopause.. 279

### E.

Experience with Cannabis Indica. Frank Dudley Beane, A. M., M. D............................ 445
Erie County Society Proceedings and Niagara University.......... 477
Eserine in Diarrhœa............ 19
Editorial Correspondence......... 88
Editorial Notes...46, 141, 185, 235, 333
Editorial Notes................. 567
Epilepsy. Floyd S. Crego, M. D., 531
Erie County Medical Society...... 127
Erie County Medical Society—Semi-annual Meeting.......... 546
Fever, Puerperal. Dr. R. L. Banta, 483
Functional and Structural Diseases, 519

### F.

PAGE.

Face Presentation, Mento-Posterior Position...................... 420
Fissure of the Anus.............. 225
Fracture of the Skull, Case of. Chas. A. Wall, M. S., M. D..... 543

### G.

Gaultheria in Rheumatism........ 136
Goodell's Mixture of the Four Chlorides..................... 138
Goodell, Wm., M. D. Clinical Lecture on Uterine Polypi...... 49
Grove, Dr. An Obscure and Interesting Case of Bright's Disease, 346

### H.

Hemorrhage, Post Partum, Remarks upon its Prevention and Treatment. W. W. Potter, M. D........................... 385
Hospital Appointments........... 428
Hopkins, H. R., M. D. Letter from .... .......... .......... 560
Headache—Pathology and Treatment of Some Forms........... 475
Heart, Organic Disease of........ 76
Hospital for Women............. 94
Hayd, Herman E., M. D., M. R. C. S. Club Foot.............. 145
Hutchinson, J. H., M. D. Apoplexy Complicated by Cirrhosis of Kidneys.................... 97
Heart, Rupture of. Dr. C. C. Wyckoff...... ................ 297

### I.

Improper Drainage and Disease.. 417
Inflammatory Processes, Local, Therapeutic Means of Affecting. 310
Influence of Benzoates of the Alkalies on the Excretion of Uric Acid......................... 26
Influence of Worry and Nervous Shock on the Uterus and its Functions..................... 554
Insanity....................331, 527

### J.

PAGE.
Jequiriti Treatment of Granular Lids. Lucien Howe, M. D .... 115

### K.

Keating, J. M., M. D. Some Observations on the Salivary Digestion of Starch by Infants........ 15
Kairin........................ 518

### L.

Late Tying of the Umbilical Cord Gives the Child More Blood .... 324
Listerine and Lithiated Hydrangea, 481
Long, B. G. Deep Abscess of the Post Cervical Region, with Sudden Death.................... 301
Lynde, U. C., M. D. Amputation of the Leg.................... 400

### M.

Measles........................ 317
Measles, Treatment of.......... 314
Medical Education in the State of New York.................... 138
Meat........................... 558
Metallic Impurities in Canned Meats ...................... 563
Metria (so-called Puerperal Fever) 70
Mynter Herman, M. D. Congenital Dislocation of both Hip Joints 406

### N.

Nephritis, Acute Diffuse. Francis Delafield, M. D.............. 318
Neurological Society, N. Y., Report 500
New Medical Bill................ 231
Niagara University, Medical Department ..................93, 184
Niagara University—Legal Opinion Regarding the Validity of its Charter...................... 435
Niagara University.............. 567
PAGE.
Nitro-Glycerine, Some Uses of. Chas. G. Stockton, M. D........ 337
Notice of Appointments.......... 382

### O.

Observation on the Salivary Digestion of Starch by Infants. J. M. Keating, M. D................ 15
Organic Disease of the Heart..... 76
Obituary........................ 142
Opium Habit, a Case of. J. W. Putnam, M. D................ 223
Opposition to the State Faculty Bill........................ 328
Obscure and Interesting Case of Bright's Disease. Dr. Grove... 346

### P.

Picric Acid as a Test for Albumen, 511
Pessaries....................... 510
Potter, W. W., M. D., Remarks upon Post Partum Hemorrhage, 385
Potter, Frank Hamilton, M. D. Selections from the Clinics of Medical Department, Niagara University.................... 456
Practical Utilization of Ozone in the Treatment of Disease. F. W. Bartlett, M. D............. 1
Puerperal Diabetes.............. 18
Peterson, Frederick, M. D. Some Interesting Autopsies 55, 105, 154 216
Pyelo-Nephritis, with Calculi in the Bladder. A. R. Davidson, M. D. 166
Professional Advertising — Is it Ethical?...................... 180
Progress of the Proposed State Faculty Bill.................. 182
Pasteur's Precaution Against the Cholera...................... 136
Pepper, Wm., M. D., LL. D. The Treatment of Long Standing Pleural Effusion..............
Prevention of Syphilis. Eli H. Long, M. D.................. 200
Putnam, J. W., M. D. A Case of Opium Habit................. 223

PAGE.
Professional Advertising......... 234
Prevention and Treatment of Puerperal Fever. F. Gaillard Thomas, M. D.......................... 302
Puerperal Fever, Treatment of.... 315
Pleurisy and Empyema in Children.......................... 273
Progress of the State Faculty Bill, 282

R.

Roberts, Dr. J. B. Surgical Delusions........... ............ 483
Reports, Buffalo Medical and Surgical Association, 36, 83, 125, 170, 229, 280, 367, 411, 461
Reports, New York Neurological Society........................ 500
Report, Erie County Medical Society......................... 464
Recurrent Attacks of Zymotic Diseases in the Same Persons.. 12
Removal of the Thyroid Gland.... 226
Report of the Commissioner of Education for the Year 1881.... 239
Rupture of Heart. Dr. C. C. Wyckoff...................... 297
Report, Annual Meeting Erie Co. Medical Society................ 367

S.

Surgical Delusions. Dr. J. B. Roberts...................... 483
Society, N. Y., Neurological, Reports.......................... 500
Storck, Dr. A Correction by..... 519
Selections from the Surgical Clinics, Medical Department Niagara University. Frank Hamilton Potter, M. D.................. 456
Stewed Fruit for the Gouty and Dyspeptic..................... 22
Sprengel: Communication in Regard to 131 Cases of Carcinoma of the Breast, Operated Upon in Volkmann's Clinic, During the Years 1874–8.................. 32
Sudden Death from Fright........ 176
PAGE.
Successful Midwifery............. 317
Scarlet Fever, Wet Pack in....... 324
Specific for Singultus............. 324
Stockton, Chas. G, M. D. Some Uses of Nitro-Glycerine........ 337
Spring Course of the Medical Colleges......................... 378
Social Medical Organization...... 381
Sponge-Tent in its Practical Application. Marcell Hartwig, M. D. 241

T.

Therapeutic Means of Affecting Local Inflammatory Processes.. 510
To Our Subscribers.............. 39
Treatment of Long Standing Pleural Effusion. Wm. Pepper, M. D., LL. D.................... 193
Training Schools for Nurses and Their Results.................. 234
Thomas T. Gaillard, M. D. Prevention and Treatment of Puerperal Fever.................... 302
Treatment of Abscess of the Neck, 313
Treatment of Measles............ 314
Treatment of Puerperal Fever.... 315
Treatment of Puerperal Septicæmia......................... 353
Treatment of Tonsillitis.......... 278
The Medical Profession.......... 374

U.

Uterine Hemostatics............. 473
Uterine Polypi. Wm. Goodell, M. D.......................... 49

V.

Volume XXIII.................. 38
Value of Ether in Collapse....... 179
Van Peyma, P. W., M. D. A Case of Angina Pectoris............ 350

W.

Wall, Chas. A., M. S., M. D. Case of Fracture of the Skull........ 543
Wyckoff, Dr. C. C. Rupture of Heart........................ 297
Wet Pack in Scarlet Fever........ 324

### TO OUR SUBSCRIBERS.

We direct attention to the bills which will be found inclosed in this number of the JOURNAL, for unpaid subscriptions. The bills also include the advance subscription for the coming volume which is in accordance with our terms. The annual subscription to the JOURNAL has been placed so low that it barely repays the cost of publication, yielding no return for the time and labor involved in editorial management. We ask our subscribers to remit the amount of their indebtedness promptly, that we may be encouraged to make needed improvements in the JOURNAL, that it may not only maintain its hitherto high reputation and be made equal if not superior to any of its metropolitan cotemporaries in the value and interest of its contents.

